# Real-Life Effectiveness and Safety of Guselkumab in Patients with Psoriasis Who Have an Inadequate Response to Ustekinumab: A 3-Year Multicenter Study

**DOI:** 10.3390/jcm13092552

**Published:** 2024-04-26

**Authors:** Matteo Megna, Anna Balato, Stefano Caccavale, Sara Cacciapuoti, Giulia Calabrese, Eugenia Veronica Di Brizzi, Luisa Di Costanzo, Raffaella Manzo, Vincenzo Marino, Rosa Valentina Puca, Francesca Romano, Oriele Sarno, Genoveffa Scotto di Luzio, Serena Lembo

**Affiliations:** 1 Section of Dermatology–Department of Clinical Medicine and Surgery, University of Naples Federico II, 80131 Napoli, Italy; 2 Dermatology Unit, University of Campania L. Vanvitelli, 80138 Naples, Italy; 3 Dermatology and Venereology, San Gennaro Hospital, 80136 Naples, Italy; 4 Department of Medicine, Surgery and Dentistry, Scuola Medica Salernitana, University of Salerno, 84084 Salerno, Italy; 5 U.O.C. Dermatologia, ASL Salerno Ospedale Tortora Pagani, 84121 Salerno, Italy; 6 Dermatology Unit, Fatebenefratelli Hospital, 20121 Benevento, Italy; 7 Department of Dermatology and Dermosurgery, AOSG San Giuseppe Moscati, 83100 Avellino, Italy; 8 Dermatology Unit, AORN “A. Cardarelli”, 80131 Naples, Italy; 9 Sant’Anna and San Sebastiano Hospital, 81100 Caserta, Italy

**Keywords:** psoriasis, biologic, guselkumab, anti-IL23

## Abstract

**Background:** Guselkumab is the first approved human IgG1λ monoclonal antibody selectively targeting the p19 subunit of IL23. Its effectiveness and safety were widely reported by clinical trials. However, these results must be confirmed in real life since its safety deals with more complicated subjects with respect to trials. Currently, real-life data on the use of guselkumab following treatment failure with ustekinumab are limited, and existing studies usually show a small cohort and/or a reduced follow-up period. In this context, the aim of our study was to evaluate the use of guselkumab in patients who previously did not respond to ustekinumab after up to 3 years of treatment. **Methods:** A multicenter retrospective study was performed. The study enrolled patients affected by moderate-to-severe plaque psoriasis undergoing treatment with guselkumab who were attending the Psoriasis Center of nine different centers in the Campania region of Italy. Demographic and clinical features were collected for each patient at baseline. Moreover, data on psoriasis severity and adverse events (AEs) were collected at each follow-up visit (week (W)16-W36-W52-W104-W156). **Results:** A total of 112 patients (70 male, 62.5%; mean age 54.8 ± 11.7 years old) were enrolled. Of these, 48 (42.9%), 34 (30.4%), and 16 (14.3%) reached 1, 2, and 3 years, respectively, of follow-up under guselkumab. A statistically significant clinical improvement was observed since W16, and sustained effectiveness was reported at each timepoint up to W156. No serious AEs were collected. Moreover, a sub analysis on the body mass index, involvement of difficult-to-treat areas, and presence of psoriatic arthritis (PsA) showed that the presence of PsA or palmoplantar psoriasis was associated with a reduced clinical improvement at W16 and W36, without differences from W52. In contrast, the efficacy of guselkumab does not seem to be affected by the BMI, involvement of fingernails, or location in the genital or scalp area. **Conclusions:** To sum up, our long-term real-life multicenter retrospective study confirmed the efficacy and safety of guselkumab following ustekinumab discontinuation up to 156 weeks of treatment.

## 1. Introduction

Psoriasis is a chronic, relapsing inflammatory skin condition affecting up to 3% of the general population [1]. Clinically, it is usually characterized by well-defined erythematous-desquamative plaques covered by whitish or silvery scales, predominantly found on elbows, knees, scalp, and the lumbar area (plaque psoriasis) [2]. However, other clinical phenotypes can be distinguished such as erythrodermic, guttate, and pustular psoriasis [2]. Several comorbidities can be associated with psoriasis [psoriatic arthritis (PsA), cardiovascular diseases, diabetes mellitus, chronic inflammatory bowel disease, psychiatric disorders, etc.]; indeed, psoriasis is considered a systemic disease [3]. Due to its high burden, psoriasis may lead to a significant worsening of patients’ quality of life, thus requiring prompt and adequate treatment [4]. In this context, mild psoriasis is often well-controlled with topical drugs (i.e., a combination of corticosteroids and vitamin D derivates), while moderate-to-severe forms usually require systemic treatments [5,6]. In these cases, conventional systemic treatments (methotrexate, cyclosporin, dimethyl fumarate, acitretin, phototherapy) are used as first-line therapies [5,6]. However, their employment may be frequently limited by low compliance, contraindications, or adverse events (AEs) [5,6]. Fortunately, advanced knowledge on psoriasis pathogenesis led to the expansion of new selective drugs, targeting interleukins (IL) involved in the psoriasis development: the biologic drugs [7]. Currently, 12 different biologics are approved for psoriasis management, targeting tumor necrosis factor (TNF)-α (adalimumab, etanercept, infliximab, certolizumab), IL12/23 (ustekinumab), IL23 (guselkumab, risankizumab, tildrakizumab), and IL17 (secukinumab, ixekizumab, brodalumab, bimekizumab) [7]. Hence, the current therapeutic scenario allows for the selection of the right drug for the right patient, thus offering the possibility of a personalized approach that has to take into account the presence of comorbidities, previous treatment failures, and patient preferences and expectations [8,9]. In this context, guselkumab is the first approved human IgG1λ monoclonal antibody selectively targeting the p19 subunit of IL23 [10]. Its effectiveness and safety have been reported by several phase III clinical trials, which also showed the superiority of adalimumab (VOYAGE1, VOYAGE2), ustekinumab (NAVIGATE), and secukinumab (ECLIPSE) [11,12,13,14]. In particular, the NAVIGATE trial investigated the effectiveness and tolerability of guselkumab in patients with moderate-to-severe plaque psoriasis who had an inadequate response to ustekinumab, showing a significant benefit from switching to guselkumab. Indeed, among the 871 patients enrolled receiving ustekinumab, 30.8% showed an inadequate response at week 16 and were randomized to guselkumab or to continue ustekinumab, showing that the proportion of patients achieving IGA 0/1 was higher for guselkumab than ustekinumab at week 28 (31.1% vs. 14.3%; *p* = 0.001) and week 52 (36.3% vs. 17.3%; *p* < 0.001) [13]. However, these results must be confirmed in real-life because safety deals with more complicated subjects with respect to trials, including higher mean of treatment failures, comorbidities, polypharmacy, or excessive polypharmacy, etc. [15].

Unfortunately, real-life data on the use of guselkumab following treatment failure with ustekinumab are limited, and existing studies usually show a small cohort and/or a reduced follow-up period. Real-world data are highly needed to confirm trial data in a more complicated setting and also because guselkumab and ustekinumab partially share their therapeutic target (the p19 subunit of IL23 vs. the p40 subunit of IL12 and IL23), as well as the frequency of subcutaneous administration (8 vs. 12 weeks), indications (psoriasis and psoriatic arthritis), and a high safety profile.

The aim of this study was to evaluate the effectiveness and safety of guselkumab in patients who previously did not respond to ustekinumab for up to 3 years of treatment.

## 2. Material and Methods

A multicenter retrospective study was performed that enrolled patients affected by moderate-to-severe plaque psoriasis undergoing treatment with guselkumab who were attending the Psoriasis Center of nine different centers in the Campania region of Italy (i.e., University of Naples Federico II, University of Campania Luigi Vanvitelli, Naples, University of Salerno, AORN “A. Cardarelli” of Naples, AOSG San Giuseppe Moscati, Avellino, “Ospedale del Mare” of Naples, Sant’Anna and San Sebastiano Hospital of Caserta, Sacro Cuore di Gesù Fatebenefratelli Hospital of Benevento, and “A. Tortora” Hospital of Pagani).

Inclusion criteria were as follows: diagnosis of moderate-to-severe plaque psoriasis assessed by a dermatologist, guselkumab treatment for at least 16 weeks, and previous failure (primary or secondary failure) of ustekinumab. Of note is that enrolled patients must have been directly switched from ustekinumab to guselkumab to be involved in the study. Exclusion criteria were as follows: patients < 18 years old; and other forms of psoriasis rather than plaque psoriasis. Data on the demographic (age, sex) and clinical features (psoriasis duration, psoriasis body location such as involvement of fingernails, palms and soles, scalp, and genital area), comorbidities, PsA history and duration (if present) or previous and current systemic psoriasis therapies (particularly focusing on ustekinumab duration and reasons of its discontinuation), and psoriasis severity using the Psoriasis Activity Severity Index (PASI) and Dermatology Life Quality Index (DLQI) were collected for each patient at baseline. Moreover, PASI and DLQI were evaluated at each follow-up visit (week 16, week 36, week 52, week 104, and week 156). Treatment-emergent AEs were assessed with physical examinations and laboratory monitoring.

Guselkumab was administered at a labeled dosage for psoriasis (100 mg as a subcutaneous injection at week 0, week 4, and every 8 weeks thereafter).

Effectiveness data were analyzed using a last observation carried forward method, where if a patient dropped out of the study, the last available value was ‘carried forward’ until the end of the treatment. The present study was conducted respecting the Declaration of Helsinki, and all patients signed an informed consent before starting the new drug, authorizing the treatment of their anonymized clinical records, as was the case of the Campania region web-based platform used for biologic drug prescription (SANIARP; www.saniarp.it).

### Statistical Analysis

Regarding statistical analysis, descriptive statistics were employed to analyze clinical and demographic data. Continuous variables are expressed as mean ± standard deviation, while categorical variables are represented by the number and proportion of patients. Statistical significance of clinical response was assessed using GraphPad Prism 4.0 (GraphPad Software Inc., La Jolla, CA, USA). The chi-square test and Student’s *t*-test were utilized to evaluate differences in values obtained at various time points of therapy for categorical and continuous variables, respectively. Statistical significance was defined as *p* < 0.05.

## 3. Results

A total of 112 patients (70 male, 62.5%; mean age 54.8 ± 11.7 years old; mean psoriasis duration 20.2 ± 10.4 years) were enrolled, matching all the inclusion and exclusion criteria. Of these, 48 (42.9%), 34 (30.4%), and 16 (14.3%) reached 1, 2, and 3 years, respectively, of follow-up under guselkumab. Hypertension was the most common comorbidity assessed (*n* = 41, 36.7%), followed by dyslipidemia (*n* = 34, 30.4%), diabetes mellitus (*n* = 20, 17.9%), cardiopathy (*n* = 10, 8.9%), and thyroid disease (*n* = 7, 6.3%). Patients’ clinical features are reported in Table 1. At baseline, mean PASI and DLQI were 10.1 ± 5.1 and 16.4 ± 6.5, respectively. Regarding the involvement of difficult-to-treat areas, 76 (67.9%), 42 (37.5%), 41 (36.6%), and 38 (33.9%) patients had the involvement of the scalp, genitalia, fingernails, and palmoplantar area, respectively, underlying the complexity of the enrolled cohort of subjects. Moreover, PsA was collected in 53 (47.3%) patients.

The majority of the patients (*n* = 99, 88.4%) were previously treated with at least one conventional systemic treatment [cyclosporine: 69 (61.6%), methotrexate: 50 (44.6%), Nb-UVB Phototherapy: 37 (33.0%), acitretin: 25 (22.3%), P-UVA: 3 (2.7%)], with only 13 (11.6%) patients naïve to these therapies.

Regarding previous treatments with biologics, 32 (28.6%), 27 (24.1%), 4 (3.6%), 2 (1.8%), and 1 (0.9%) patients failed adalimumab, etanercept, secukinumab, infliximab, and golimumab, respectively, whereas 3 (2.7%) subjects failed apremilast.

All of the patients previously failed ustekinumab (mean duration of therapy: 47.7 ± 31.9 months), discontinuing treatment mainly for secondary loss of efficacy (*n* = 98, 87.5%), followed by AEs (*n* = 7, 6.3%), including COVID-19 infection (*n* = 1, 0.9%), and hepatitis C infection (*n* = 1, 0.9%), primary inefficacy (*n* = 4, 3.6%), and PsA worsening (*n* = 3, 2.7%).

**Figure 1:**
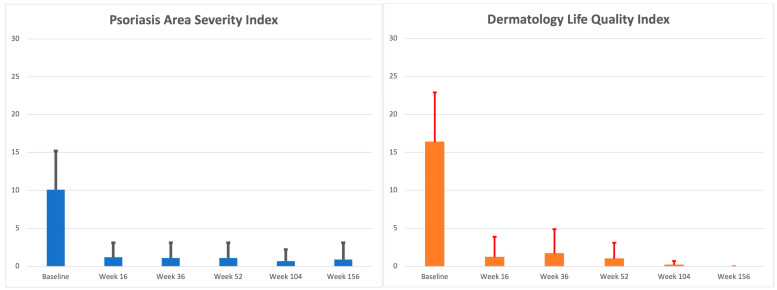
Mean PASI and DLQI assessment (mean value and standard deviation) at baseline, week 16, week 36, week 52, week 104, and week 156.

**Figure 2:**
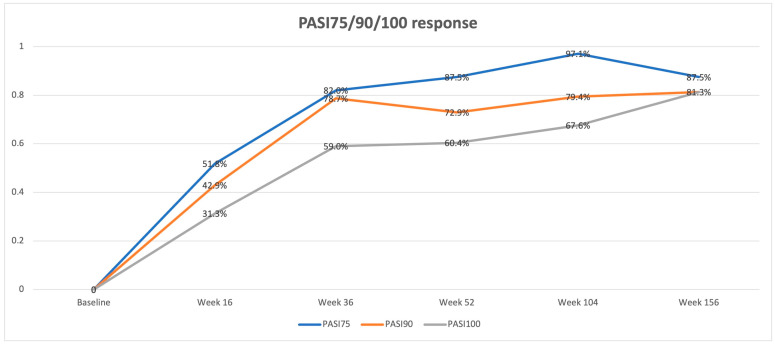
Percentage of patients achieving PASI75/90/10 response at week 16, week 36, week 52, week 104, and week 156.

Wash out from the end of ustekinumab to the start of guselkumab therapy was not required. The baseline clinical evaluation showed a mean PASI of 10.1 ± 5.1 and a mean DLQI of 16.4 ± 6.5, depicting a severe disease with significant QoL impairment. Both scores significantly reduced at week 16 (PASI: 1.2 ± 1.9; DLQI: 1.2 ± 2.7, *p* < 0.0001 for both), showing sustained effectiveness at each timepoint up to week 156 (PASI: 0.9 ± 2.2; DLQI: 0). PASI and DLQI trends are reported in Table 1 and Figure 1. The percentage of PASI75/90/100 responses at each follow-up is reported in Figure 2.

Regarding safety, no cases of serious AEs, injection site reactions, candida, major cardiovascular events, or malignancy were collected in our study. 

Of interest, a sub analysis on BMI, involvement of difficult-to-treat areas, and presence of PsA was performed to identify possible predictive response factors. Regarding the presence of PsA, although PASI at baseline was comparable among patients with (*n* = 53, 47.3%) or without (*n* = 59, 52.7%) PsA (10.4 ± 5.8 vs. 9.8 ± 4.4), patients without PsA experienced better skin improvement at week 16 (PASI: 0.8 ± 1.5 vs. 1.6 ± 2.3, *p* < 0.05) and 36 (PASI: 0.8 ± 1.6 vs. 1.3 ± 2.4, *p* < 0.001) compared with patients with PsA. Of note, no differences were found from week 52. 

Similarly, we found that patients with the involvement of the palmoplantar area (*n* = 38, 33.9%) had a reduced improvement of PASI compared with patients without palmoplantar area involvement (*n* = 74, 66.1%) at week 16 (1.7 ± 2.2. vs. 0.9 ± 1.7, *p* < 0.05) and week 36 (1.7 ± 2.3 vs. 0.7 ± 1.8, *p* < 0.05), as compared with the baseline (9.1 ± 4.0 vs. 10.6 ± 5.5). 

Finally, the efficacy of guselkumab does not seem to be affected by BMI or the involvement of fingernails or the genital or scalp area. 

## 4. Discussion

Psoriasis management may be challenging, particularly for moderate-to-severe forms of the disease [16]. However, several strategies are emerging such as the use of antioxidants as a potential therapeutic strategy in the treatment of psoriasis [17]. Despite the introduction of biologics completely changing treatment scenario, more studies are required to better understand when and which biologic to choose during treatment switching [18]. In this context, more and more real-life data are needed since clinical practice is quite different from trials as the patients may vary, including several clinical and individual features which may be excluded from clinical trials [15]. Indeed, it should be noted that specific inclusion and exclusion criteria do not allow the generalizability of the results from trials, whereas there are some complicated patients (comorbidities, age, previous psoriasis treatments, concomitant medications, etc.) which are often under-considered in trials but are present in the real-world setting [15]. Thus, even though the NAVIGATE study has already shown a significant benefit from switching to guselkumab in patients with an inadequate response to ustekinumab, further real-world evidence is mandatory.

In this context, we performed a 3-year multicenter study in the Campania region of Italy with the aim of evaluating the effectiveness of guselkumab in patients previously treated with ustekinumab. A total of 112 patients were enrolled in our study. Of these, 48 (42.9%), 34 (30.4%), and 16 (14.3%) achieved at least 1, 2, and 3 years of treatment with guselkumab, respectively. Globally, secondary loss of efficacy (*n* = 98, 87.5%) was the most common cause of ustekinumab discontinuation, followed by primary inefficacy (*n* = 4, 3.6%), AEs (*n* = 7, 6.3%), including COVID-19 infection (*n* = 1, 0.9%), and hepatitis C infection (*n* = 1, 0.9%). Finally, 3 (2.7%) patients discontinued ustekinumab because their PsA worsened.

It should be noted that the high mean duration of ustekinumab treatment of 47.7 ± 31.9 months was based on data that frequently lacked real-life examples of switching from ustekinumab to guselkumab. 

In our experience, a statistically significant improvement in both PASI and DLQI was collected since week 16, compared with the baseline (PASI: 1.2 ± 1.9 vs. 10.1 ± 5.1; DLQI: 1.2 ± 2.7 vs. 16.4 ± 6.5; *p* < 0.0001 for both), with 51.8%, 42.9%, and 31.3% of patients achieving PASI75/90/100 response, respectively. Clinical improvement was confirmed at each follow-up visit up to week 156 (PASI: 0.9 ± 2.2; DLQI: 0; PASI75/90/100: 87.5%/81.3%/81.3%). Therefore, guselkumab showed a significant maintenance of treatment response over time (Figure 1 and Figure 2). It should be underlined that the guselkumab reached these results even if about half of our population (*n* = 53, 47.3%) was affected by PsA and when the difficult-to-treat areas were frequently involved (scalp: 67.9%; genitals: 37.5%; fingernails: 36.6%; palms or soles: 33.9%). In this context, we noticed that patients without PsA and without the involvement of the palmoplantar area experienced better skin improvement at week 16 and week 36 compared with patients with PsA and patients with palmoplantar area involvement, respectively. However, BMI and involvement of fingernails and the genital or scalp area did not seem to affect the therapeutic outcome. Finally, no cases of serious AEs were collected, confirming the safety of guselkumab.

Hence, our results confirmed the effectiveness of guselkumab reported by clinical trials but in a more complicated setting (i.e., higher PsA prevalence and difficult-to-treat area involvement).

In the NAVIGATE study, among the 135 patients who were switched from ustekinumab to guselkumab, 51.1% reached PASI90 at week 16 and 20.0% reached PASI100 at week 52, significantly higher than the 133 patients who continued ustekinumab (PASI90: 24.1%, *p* < 0.001; PASI100: 7.0%, *p* = 0.003) [13].

Another recently published phase IV clinical trial (COBRA) showed that 35.9% of subjects receiving guselkumab following ustekinumab discontinuation achieved a PASI100 response at week 16 [19]. However, the main aim of the trial was comparing brodalumab and guselkumab following ustekinumab discontinuation, and the results showed a higher percentage of patients receiving brodalumab (53.4%) achieved a PASI100 response at week 16 [odds ratio (OR) 2.05; 95% confidence interval (CI) 0.95, 4.44; *p* = 0.069] [19]. 

Regarding real-life studies, Berenguer-Ruiz et al. reported the first real-life results of a multicenter study specifically investigating the effectiveness of switching from ustekinumab to guselkumab. In particular, the mean age of patients was comparable with our cohort (53.7 ± 13.5 years vs. 54.8 ± 11.7 years), whereas psoriasis duration was higher than ours (26.0 ± 12.0 years vs. 20.2 ± 10.4 years) [20]. The main reasons for ustekinumab discontinuation were insufficient skin and/or joint response (84.4%) and suboptimal response (16.6%). At baseline, the mean PASI was lower than ours (7.6 ± 6.5 vs. 10.1 ± 5.1) [20]. Globally, 96.5%, 72%, and 29% of patients reached PASI <5, PASI<2, and PASI100 responses at week 24, respectively [20]. However, preliminary data on the effectiveness of guselkumab following ustekinumab administration were reported by Fougerousse in their retrospective, real-life study with the aim of evaluating the short-term effectiveness and tolerance of guselkumab for psoriasis under real-life conditions [21]. The authors showed that among the 63 patients who received guselkumab after ustekinumab, 41.3% achieved PASI90 response at week 16, in line with our results (PASI90 at week 16; 42.9%) [21].

The longer real-life study investigating the effectiveness of guselkumab following ustekinumab was reported by Gargiulo et al. In their multicenter experience, 233 subjects were enrolled (54.3 ± 13.3 years, mean psoriasis duration 24.2 ± 13.4 years) [22]. Despite the results in terms of PASI75/90/100 response at week 16 being slightly higher than ours (76.4%/46.8%/38.8% vs. 51.8%/42.9%/31.3%), they become comparable at week 104 (97.1%/79.4%/67.6% vs. 90%/73.8%/60%) [22]. Moreover, the safety profile was comparable (i.e., no significant safety findings were reported in both studies) [22]. Of interest, Gargiulo et al. also investigated difficult-to-treat areas and showed that patients with their involvement were less likely to achieve PASI90 and PASI100 at week 16, as well as obese patients had significantly lower rates of PASI75 and PASI ≤2 at week 52 [22]. However, at week 104, comparable responses were observed among all patients’ subgroups. In our experience, we found that patients with PsA or with the involvement of the palmoplantar area had a significantly reduced improvement of PASI at week 16 and week 36 [22]. These differences were not confirmed from week 52; however, the results from week 52 may be influenced by the reduced numerosity of the cohort who reached longer follow-ups [22]. In contrast, compared to Gargiulo et al., we reported that the use of guselkumab in ustekinumab failure patients does not seem to be affected by the BMI, involvement of fingernails, genital or scalp [22]. 

Finally, other experiences confirmed the efficacy of guselkumab following ustekinumab interruption despite having a limited cohort of subjects [23,24,25]. 

It should be noted that a recently published multicenter, observational, and retrospective study showed that guselkumab use was not impacted by previous biologics, with a treatment survival of 92.7% of patients at 130 weeks among subject previously receiving an anti-IL17 (*n* = 29), compared with 100% and 92.1% of patients previously treated with anti-TNFα, (*n* = 29) and ustekinumab (*n* = 45) [26].

To the best of our knowledge, our study was the longest (156 weeks) real-life experience investigating the effectiveness of guselkumab following ustekinumab discontinuation. Despite its limitations and retrospective design, our data showed that switching from ustekinumab to guselkumab does not affect the effectiveness of the anti-IL23. These findings suggest that the previous inhibition of the IL23 p40 subunit does not affect the inhibition of the IL23 p19 one, even in subgroups of complicated patients with frequent involvement of difficult-to-treat areas and high PsA prevalence (47.3%). Palmoplantar involvement and the presence of PsA seem to be linked to a slightly reduced response up to 52 weeks of treatment.

Certainly, further clinical real-life studies would corroborate our results.

### Strengths and Limitations of the Study

The statistical methods, the higher percentage of patients with PsA or involvement of difficult-to-treat areas, and the considerable duration of treatment with ustekinumab are the main strengths of our work.

The retrospective nature of the study, the relatively small size of our cohort, and the limited number of patients achieving 3 years of follow-up are the main limitations, reducing the generalizability of our results.

## 5. Conclusions

Our long-term real-life multicenter retrospective study confirmed the efficacy and safety of guselkumab following ustekinumab discontinuation up to 156 weeks of treatment. However, further studies are required to confirm our results. 

## Figures and Tables

**Table 1:** Patients’ feature at baseline (Week 0) and psoriasis assessment at baseline, week 16, week 36, week 52, week 104, and week 156. Nb-UVB (Narrow band–Ultraviolet B). P-UVA: Psoralen-Ultraviolet A. PASI: Psoriasis Activity Severity Index. DLQI: Dermatology Life Quality Index.

**Number of patients**				112		
**Sex:**						
Male				70 (62.6%)		
Female				42 (37.5%)		
**Mean age** *(years)*				54.8 ± 11.7		
**Mean duration of psoriasis** *(years)*				20.2 ± 10.4		
**Psoriatic Arthritis**				53 (47.3%)		
**Difficult-to-treat areas involvement**						
Scalp				76 (67.9%)		
Palms or soles				38 (33.9%)		
Genital				42 (37.5%)		
Fingernails				41 (36.6%)		
**Comorbidities:**						
Hypertension				41 (36.7%)		
Dyslipidemia				34 (30.4%)		
Obesity				27 (24.1%)		
Diabetes				20 (17.9%)		
Cardiopathy				10 (8.9%)		
Thyropathy				7 (6.3%)		
**Previous systemic treatments (conventional and small molecules):**						
Methotrexate				50 (44.6%)		
Cyclosporine				69 (61.6%)		
Nb-UVB Phototherapy				37 (33.0%)		
P-UVA				3 (2.7%)		
Acitretin				25 (22.3%)		
Apremilast				3 (2.7%)		
Naïve				13 (11.6%)		
**Previous biologic treatments:**						
Anti-TNFα						
Adalimumab				32 (28.6%)		
Etanercept				27 (24.1%)		
Infliximab				2 (1.8%)		
Golimumab				1 (0.9%)		
**Anti-IL12/23**				112 (100%)		
**Anti-IL17**						
Secukinumab				4 (3.6%)		
	**BASELINE**	**WEEK 16**	**WEEK 36**	**WEEK 52**	**WEEK 104**	**WEEK 156**
**Mean PASI**	10.1 ± 5.1	1.2 ± 1.9	1.1 ± 2.0	1.1 ± 2.0	0.7 ± 1.5	0.9 ± 2.2
**MeanDLQI**	16.4 ± 6.5	1.2 ± 2.7	1.7 ± 3.2	1.0 ± 2.1	0.2 ± 0.5	0
**PASI75**	/	51.8%	82.0%	87.5%	97.1%	87.5%
**PASI90**	/	42.9%	78.7%	72.9%	79.4%	81.3%
**PASI100**	/	31.3%	59.0%	60.4%	67.6%	81.3%

## Data Availability

Data that support the findings of this study are available from the corresponding author, upon reasonable request.
